# Diet Mixing and Supplementation Present an Opportunity to Increase the Use of Encroaching Woody Plants by Goats

**DOI:** 10.3390/ani13223509

**Published:** 2023-11-14

**Authors:** Piet Monegi, Ntuthuko Raphael Mkhize, Purity Thobekile Masondo, Khanyisile Rebecca Mbatha, Dibungi Luseba, Julius Tlou Tjelele

**Affiliations:** 1 Agricultural Research Council, Animal Production, Range and Forage Sciences, Irene, Pretoria 0062, South Africa; 2 Department of Agriculture and Animal Health, University of South Africa, Florida, Roodepoort 1709, South Africa; mbathkr@unisa.ac.za; 3 Animal and Poultry Science, School of Agriculture, Earth and Environmental Sciences, University of KwaZulu-Natal, Scottsville, Pietermaritzburg 3209, South Africa; 4 Department of Animal Science, Faculty of Science, Tshwane University of Technology, Staatsartillerie Road, Pretoria West, Pretoria 0001, South Africalusebad@tut.ac.za (D.L.)

**Keywords:** secondary compounds, nutrient–toxin interactions, macronutrients, feed intake

## Abstract

**Simple Summary:**

Without strategies to increase the use of chemically defended trees as forage, feeding 9 billion people by 2050 becomes impossible. In response to the challenge of trees encroaching on grasslands and the growing demand for meat, this study conducted experiments to support sustainable solutions for future animal protein needs. The research focused on enhancing goat consumption of tannin-defended woody plants through supplementing their diets with nutrients and polyethylene glycol. It also explored the impact of diverse diet ingredients and plant species on goats’ intake of chemically defended woody plants. The results highlight the effectiveness of incorporating various plant species and nutrient-rich options in goat diets to help them consume chemically defended woody plants. This research provides valuable insights for sustainable livestock farming, enabling us to meet the increasing meat demand while conserving our evolving ecosystems.

**Abstract:**

Along with the woody plant expansion that is predicted to continue at the expense of the grassy layer is the increasing societal demand for animal protein and livestock products. Unless concerted efforts by land users, ecologists, and animal scientists are made to increase the utilization of trees and shrubs as forage, it will be impossible to meet future demand for meat and meat products. We conducted two short-term pen experiments to determine the effects of (1) supplementation with polyethylene glycol (PEG-a polymer purported to bind and neutralize the negative effects of tannins), a high-protein source (soybean meal), and a high-energy source (yellow maize grain) and (2) diet mixing (single-species vs. multispecies diets) on the intake of condensed tannin-rich woody plants (i.e., *Searsia lancea*, *S*. *pyroides,* and *Euclea crispa*) by goats. While all three forage species were used in the diet mixing experiment (Exp. 2), only *E. crispa* was used in the supplementation experiment (Exp. 1). Supplementing goats with energy- and protein-rich sources significantly increased the intake of *E*. *crispa* (*p* < 0.05), 713.4 g ± 13.5 and 760 g ± 28.9, respectively, whereas those on the control diet maintained their intake at 540.32 g ± 11.2. Although PEG tended to increase the consumption of *E. crispa* by goats, the observed increase was not significant (*p* > 0.05) from that observed in other treatments. In the diet mixing experiment, goats offered a combination of all three forage species attained substantially higher dry matter intakes compared to the goats offered these species individually (*p* < 0.05). While longer-term field experiments are needed in the African savannas, we postulate from the current results that management strategies that provide animals with (1) a variety of species in the diet vs. monocultures and (2) a combination of nutrient-rich and tannin-rich species may improve the ability of goats to consume chemically defended woody plants.

## 1. Introduction

Along with the woody plant expansion that is predicted to continue at the expense of the grassy layer, especially in arid and semiarid rangelands [1,2], is the increasing societal demand for animal protein and livestock products [3,4]. By 2030, the numbers of cattle, buffaloes, sheep, and goats in the developing world alone are projected to exceed those of the entire world [5]. To feed these additional numbers, about 3.2 billion tons of additional animal feed per year will be needed [6]. Unless concerted efforts by land users, ecologists, and animal scientists are made to increase the utilization of trees and shrubs as forage, it will be impossible to meet future demand for meat and meat products. This demand will be exacerbated by the expected escalation in human population, which is expected to reach a high of 9 billion by 2050 [7]. Although woody plants and shrubs are already used as an important source of nutrients for goats in arid and semi-arid environments, their intake is hugely limited by the widespread presence of plant secondary metabolites (PSMs) [8,9]. For efforts towards the safe and sustainable use of woody species as forage to yield any tangible results, we need to improve our understanding of the mechanisms through which herbivores such as goats cope with and/or overcome the negative effects of PSMs.

It is now well established in behavioral ecology that mammalian herbivores evolved some behavioral and physiological coping strategies against plant defenses [8,10]. These strategies are largely explained in terms of the detoxification limiting theory [11], which predicts, among others, that herbivores will increase the variety of plant items in their diets to possibly improve their ability to consume chemically defended plants. According to this prediction, herbivores consume more PSM-rich plants with a wide range of chemical diversity because the detoxification of dietary toxins can be spread over many metabolic pathways [12]. The logic behind this is that different PSMs are less toxic when consumed as a dilute mixture than when one PSM is consumed in larger amounts [11]. Another prediction that was developed from this theory predicts herbivores to increase the intake of plants containing PSMs as long as their capacity to neutralize and excrete these toxins is not exceeded [13,14,15]. That is because processes such as the synthesis of detoxification enzymes, supply of carbohydrate and amino acid precursors for conjugation/excretion, and maintenance of the acid/base balance to compensate for acidic end-product formation can all deplete the nutrients [16,17]. Thus, detoxification processes reduce the energy and proteins that otherwise would be available to animals for their growth, maintenance, or reproduction, with serious implications for herbivore productivity in chemically defended plant systems.

Even though the effects of diet mixing [18,19,20,21,22] and nutrient supplementation [16,23] on the intake of PSM-rich plants have previously been shown elsewhere, very little evidence (if at all) is based on arid/semiarid African savannas. Most of what is known so far on diet mixing is based on studies in North American and Mediterranean ecosystems [18,19,20,21,22] that are predominantly endowed with nitrogen-based PSMs [18,24]. Given that African savanna woody plants are predominantly defended by carbon-based PSMs [25,26,27], such as condensed tannins, the detoxification limitation theory may not adequately explain how PSMs mediate plant–herbivore interactions in African savanna ecosystems. Condensed tannins (CTs) are generally not toxins [28], but digestibility reducers [23,29] that are structurally too large to be absorbed, detoxified, and excreted through the liver [28,30,31]. Moreover, while many studies have shown the use of chemicals such as polyethylene glycol (PEG) to boost the herbivores’ tolerance for CTs, it is still to be determined how PEG’s effectiveness compares to that of nutrients (i.e., energy and protein) supplementation.

Using indigenous goats as model herbivores, we conducted two pen experiments to determine the effects of (1) supplementation with PEG, a high-protein source (soybean meal), and a high-energy source (yellow maize grain) and (2) diet mixing (single-species/monoculture vs. multispecies diets) on the intake of CT-rich woody plants. Based on the literature, we expected the diversity of plant species in the diet to allow for a higher intake than diets based on a single species. Secondly, we expected supplemental nutrients and PEG to increase the consumption of a CT-rich plant, with PEG-treated goats achieving the highest intake. Recent studies have shown goats to possess some salivary tannin-binding proteins that effectively neutralize tannins found in dietary woody plant species [32,33]. Thus, while nutrient-supplemented goats have to await post-ingestive feedback before increasing their intake of CT-rich plants [34], PEG-supplemented goats should incur less costs for nutrients to neutralize CTs. Moreover, there is a possibility that nutrient supplementation may induce satiety for energy and proteins [35] in ways that cause them to stop eating quicker than PEG-treated animals.

## 2. Materials and Methods

### 2.1. Study Area

This study was conducted at the Agricultural Research Council (ARC) Roodeplaat Experimental Farm, Gauteng Province, South Africa (28°19′ E, 25°35′ S). The farm’s natural vegetation component, which is used for livestock production and wildlife, covers over 2100 ha. The vegetation type of the farm is classified as the Marikana Thornveld [36]. The average annual precipitation is 687 mm, with the majority of it falling during the austral summer (November to March). The daily maximum temperature in summer ranges between 20–29 °C, while the minimum winter temperature can decrease to 2–16 °C.

### 2.2. Study Plants and Animals

Three plant species, namely, *Searsia lancea*, *Searsia pyroides*, and *Euclea crispa,* were used in this study. These plants occur abundantly in the study area in particular and are the common encroaching plant species in African savannas in general. All three plant species are known to be highly defended by CTs [37]. While *S. pyroides* is deciduous, *S. lancea* and *E. crispa* are evergreen species that are available throughout the year. We used [38]’s nomenclature for tree species.

The study animals, goats (*Capra hircus*), are important and common domestic mixed feeders in African savannas [39]. They have the character of surviving on harsh and degraded rangelands [40]. Woody plants are an important constituent of the diet of goats, though they are mixed feeders. Goats usually graze during the wet season and switch to browsing during the dry season. Non-descript indigenous goats were used in this study [41]. These goats are characterized by their small body size, slow growth rates, low milk yield, and low carcass weight.

### 2.3. Experimental Designs

After three weeks of conditioning periods, the two feeding experiments were conducted, and the experiments ran for 14 days each. The first experiment compared the browse intake of goats that were offered the three plant species separately to the goats that were offered a combination of all three species at once. The second experiment compared the browse intake of goats that were supplemented with maize grain, soybean meal, and PEG with the control group. In both experiments, 24 indigenous female goats aged between 1.5 and 2 years with a mean body weight of 26.6 kg ± 0.51 (mean ± SEM) were used. All goats were dewormed before the experiment. Animals were individually placed in 2 × 2 m pens to feed them separately so that the daily feed intake for each goat could be recorded. They were offered fresh clean water ad libitum throughout the experiments. Animals were weighed one day before the experiment and on the last day of the experiment to monitor the effect of the experiment on their health and well-being. In addition, the diet provided in this study was carefully monitored to ensure that aflatoxin levels were well below the established safety limits for animal feed. This precautionary measure was taken to safeguard the animals’ health and welfare. During the habituation period, goats were provided with grass and branches of the study species to familiarize them with these plants and the feeding conditions.

### 2.4. Experiment One

The 24 goats were randomly divided into four groups of six animals each. Each group was fed one of the four diets (diet one: *S. lancea*; diet two: *S. pyroides*; diet three: *E. crispa;* and diet four: a combination of all three species (*S. lancea*, *S. pyroides*, and *E. crispa*)). Diet four comprised equal weights of *S*. *lancea*, *S*. *pyroides*, and *E*. *crispa* leaves mixed. Each animal was offered ad libitum access to the experimental diet allocated to it for three hours between 9:00 am and 12:00 pm daily. Animals received fresh *S*. *lancea*, *S*. *pyroides*, and *E*. *crispa* leaves that were collected every morning before feeding. The experimental diet for each animal was weighed before and after feeding. Daily browse intake was calculated as the difference between the feed-in and feed-out of leaves. Feed intake was reported on a dry matter basis. To calculate the dry matter feed intake of the diets, fresh leaves of the study plants were weighed and oven-dried. Plant samples were oven-dried at 70 °C for 72 h. After the diets were removed from the oven, they were weighed again to calculate the dry matter of the diets. All animals received a maintenance diet of *Medicago sativa* ad libitum between 14:30 and 17:00, after which all refusals were collected and no other feed was offered until the next day. This was carried out to prevent goats from feeding overnight and to stimulate the voluntary intake of the experimental diets the following morning.

### 2.5. Experiment Two

Twenty-four goats were divided into four groups of six goats each. Goats were selected randomly and assigned to different treatments. During the experiment, goats in treatment (1) were given 100 g maize gain + leaves of *E. crispa*. Goats in treatment (2) were given 100 g soybean meal + leaves of *E*. *crispa*. In treatment (3), goats were given 40 mL polyethylene glycol + leaves of *E. crispa*, while in treatment (4) were given *Euclea crispa* only. Goats received fresh *E*. *crispa* leaves ad libitum. Soybean meal contained 51.8% DM crude protein (CP) and 18.2 MJ/kg DM digestible energy [42], while maize grain contained 10.5% DM CP and 16.2 MJ/kg DM digestible energy [37,43]. Thus, both supplements are high in energy, but maize grain is much lower in CP. Every morning, goats received their respective treatments. It took around 20 min for the goats to finish consuming the maize, soya beans, and PEG every day. The 40 mL PEG given to treatment (3) goats was based on the preliminary results. The amounts of maize and soya provided were small enough to prevent gut fill. *E. crispa* leaves were weighed and given to animals every morning. Leaves left in each pen were weighed and recorded each day in the afternoon. This was performed to get the daily feed intake of each goat. The daily browse intake was calculated as the difference between feed in and feed out of leaves and was converted to dry matter as described above.

### 2.6. Chemical Composition

Each day during the experiment, a random grab sample of each diet was taken and bulked in a sealed bag, pending analysis. The experimental diets were analysed for crude protein (CP) using the Kjeldahl block digestion method [44] and neutral detergent fibre (NDF) using the tector fibertec system [45]. Acid detergent fibre (ADF) and acid detergent lignin (ADL) were analysed using heat treatment of the samples with sulphuric acid containing cetyltrimethyl ammonium bromide [46].

### 2.7. Data Analyses

Prior to analysis, the normality and homoscedasticity of data were checked using Shapiro–Wilk and Levene’s tests, respectively. In both experiments, general linear models were used. Diets, in the first experiment, and treatments, in the second experiment, were used as fixed factors. Dry matter browse intake was a dependent variable. Each goat (*n* = 24) was considered as an experimental unit in both experiments. Differences among means were considered significant at the 5% level and SPSS version 22 (2013) was used for all data analyses. Assumptions of normality distribution of data and homogeneity of variances were met by the data.

## 3. Results

**Figure 1:**
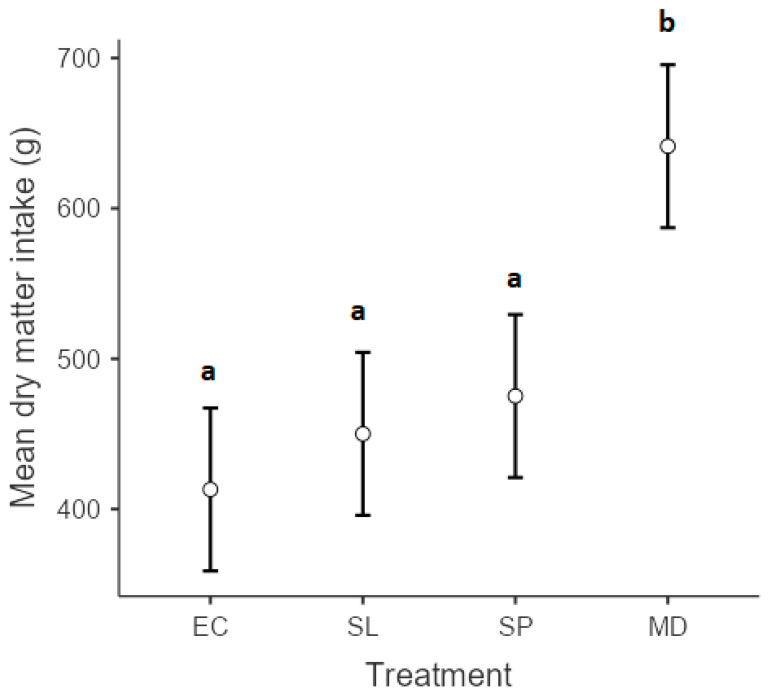
Dry matter intake (g) of *Euclea crispa* (EC), *Searsia lancea* (SL), and *Searsia pyroides* (SP) fed to goats individually and all (MD) as one meal. Different superscripts indicate significantly different means according to Scheffé’s test (*p* < 0.05).

Overall browse intake varied among experimental diets (*p* < 0.01), with goats offered a combination of *S. lancea*, *S pyroides*, and *E. crispa* attaining the highest dry matter intake (641.4 ± 34) compared to the goats offered *S. lancea* (450.1 ± 25.2), *S pyroides* (475.2 ± 24.7), and *E. crispa* (413.1 ± 23.3) as individual diets (Figure 1). Although the dry matter intake of *E. crispa* tended to be lowest, no significant differences were observed among goats offered diets 1, 2, and 3 individually. Results for the chemical composition of the study plants are presented in Table 1.

**Figure 2:**
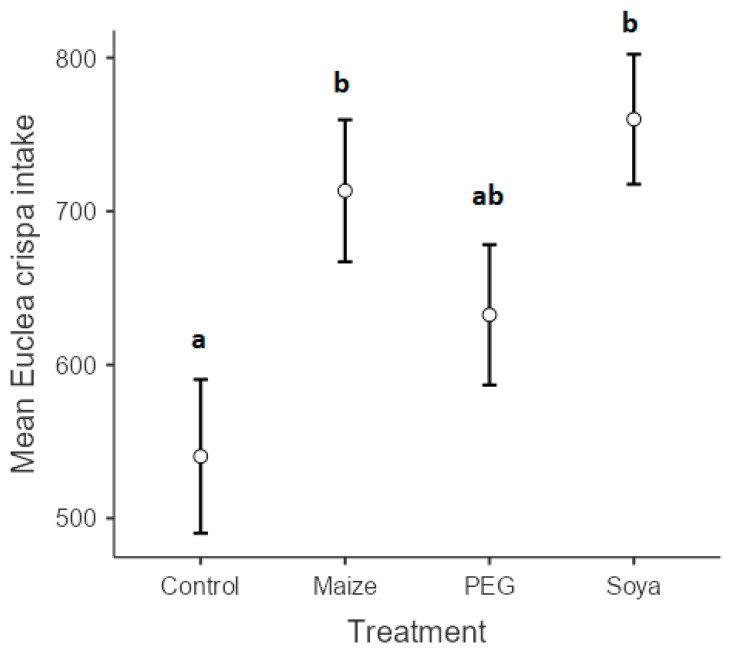
Mean *Euclea crispa* intake (g) of goats that were supplemented with polyethylene glycol, maize grain, and soybean meal. Different superscripts within a column indicate significantly different means according to Scheffé’s test (*p* < 0.05).

The dry matter intake of *E*. *crispa* was significantly influenced by the treatments (*p* < 0.001) (Figure 2). Although there was a clear tendency for PEG to increase intake of *E. crispa* (632.5 g ± 27.3), the huge variation among individual goats made it impossible for the differences to be statistically significant. Despite huge variations among individuals, supplementation with either maize grain or soybean meal increased goats’ intake of *E. crispa*, 713.4 g ± 13.5 and 760 g ± 28.9, respectively. Goats that received *E*. *crispa* (control) only recorded the lowest intake at 540.32 g ± 11.2.

## 4. Discussion

The significantly higher intake of mixed forage relative to intakes of single browse species by goats is in line with our predictions. These results are also in line with the notion that animals select various food items to match their nutrient requirements whilst avoiding the over-ingestion of plant secondary metabolites [47,48]. These results are consistent with those from other studies that showed a mixed diet to allow herbivores to increase their intake of different PSMs [18,20]. The increase in dry matter browse intake by goats that were exposed to diets with diverse species has positive implications for animal production. For example, some of the protein may escape the rumen in the form of protein–tannin complexes for absorption in the small intestines (i.e., bypass protein) [49]. Increased-tannin browse material may also have positive benefits by reducing internal parasite burden [50]. These results demonstrate that variety in the diet can be used as a tool to increase the utilization of condensed tannin-rich woody plants in African savannas. These results also suggest that monoculture foraging systems may limit goats’ ability to physiologically deal with plant secondary metabolites in their diet, which manifests behaviourally through animals limiting their intake of chemically defended plants.

In line with our prediction, supplementing goats with energy- and protein-rich sources increased intake of the tannin-rich *E. crispa*. Similar studies in field experiments found that supplemental energy and protein increase the consumption of chemically defended shrubs in the same study area [51]. Banner et al. [14] found that energy from supplemental barley increased the intake of sagebrush by lambs that were fed a basal ration of alfalfa pellets that are high in protein. Dziba et al. [15] concluded that supplemental macronutrients increase the use of sagebrush by ewes. The only difference between the current study and the two studies on sagebrush is that sagebrush contains toxic terpenes [15], while condensed tannins chemically defend *E. crispa* in African savannas. Moreover, these studies were on sheep while the current one is on goats. The three studies cited above were conducted in the field, while the current one was in a pen setup.

Despite large variations among individual animals, these results indicated clearly that supplementing goats with maize grain and soybean meal increased the dry matter intake of *E. crispa*. This observation was against our expectations and may be explained in terms of the nutrient composition of the two supplements. Though soybean is high in CP and maize is high in energy, they probably both have enough quantities of these nutrients to help the animals deal with or neutralize the negative effects of condensed tannins [9]. The current understanding is that tannins bind with dietary proteins, reducing their digestibility [29,52]. Our prediction was based on the expectation that proteins from soybean meal would replace the protein lost by animals via the secretion of salivary protein [53]. This is because goats are known to possess tannin-binding salivary proteins that help them prevent the negative effects of tannins on protein digestion [32]. These salivary proteins are known to bind readily to dietary tannins in the oral cavity to prevent the tannins from interacting with other proteins [9]. In this study, it was expected that the condensed tannins in *E. crispa* would cost goats some salivary protein, and thus the protein-rich supplement would help the animal make up for that protein loss. Such a compensation would in turn allow the animal to consume more *E. crispa* leaves. However, these results suggest that both energy-rich and protein-rich supplements probably contained enough energy or protein for the goats to increase their intake of a tannin-rich plant.

The prediction that PEG would increase the consumption of *E. crispa* by goats was not fully supported by the results. The PEG is generally known to irreversibly bind to tannins in ways that prevent the negative effects of tannins on nutrient digestion [13,52]. These results can also be attributed to the short-term nature of the experiment, which might have allowed for huge variations among individual animals. From these results, we postulate that PEG requires a longer period than 14 days to impose significant effects on the intake of tannin-rich plants. These results contradicted those of Villalba et al. [13], who reported that PEG substantially increased the intake of food high in tannins. Another study by Rogosic et al. [21] found supplemental PEG to increase the intake of *Pistacia lentiscus* (a tannin-rich Mediterranean shrub). The later study further concluded that PEG had a greater influence on sheep than on goats, which might explain the current results. A study that was conducted in the same area as the current one found PEG to not only increase the intake of tannin-rich plants but also increase the amount of time goats spend browsing [48]. Nonetheless, the findings from the current study were consistent with [54], who reported that PEG supplementation did not influence *Chiliotrichum diffusum* (an unpalatable shrub) intake by ewes.

## 5. Conclusions

In conclusion, mixed diets increase the intake of tannin-rich plants. Supplementing goats with energy-rich and protein-rich sources increases the intake of tannin-rich plants by goats. Energy- and protein-rich sources did not differ significantly in influencing the intake of a tannin-rich plant. Lastly, PEG did not influence the intake of tannin-rich species by goats. This work has serious implications for goat and rangeland productivity in savannas. The current study shows how strategies that improve herbivores’ ability to consume woody plants can improve the efficiency of the vegetation management plan, as well as the nutrition and welfare of livestock.

## Figures and Tables

**Table 1:** Chemical composition of *S*. *lancea*, *S*. *pyroides*, and *E*. *crispa*. CP = crude protein, NDF = neutral detergent fibre, ADF = acid detergent fibre, and ADL = acid detergent lignin.

Parameters	*S*. *lancea*	*S*. *pyroides*	*E*. *crispa*
CP (%)	9.5 ± 0.6	12.1 ± 0.8	7.5 ± 0.2
NDF (%)	42.3 ± 0.6	44.4 ± 1.1	34.8 ± 0.7
ADF (%)	22.1 ± 0.5	28.2 ± 0.4	25.3 ± 0.5
ADL (%)	7.5 ± 1.0	13.3 ± 1.5	13.5 ± 0.6

## Data Availability

The data that support this study will be shared upon reasonable request to the corresponding author.
